# Lay Beliefs About Interaction Quality: An Expertise Perspective on Individual Differences in Interpersonal Emotion Ability

**DOI:** 10.3389/fpsyg.2020.00277

**Published:** 2020-02-25

**Authors:** Marcus G. Wild, Jo-Anne Bachorowski

**Affiliations:** Department of Psychology, Vanderbilt University, Nashville, TN, United States

**Keywords:** social interactions, emotion, expertise, affect induction, lay beliefs

## Abstract

Social interactions have long been a source of lay beliefs about the ways in which psychological constructs operate. Some of the most enduring psychological constructs to become common lay beliefs originated from research focused on social-emotional processes. “Emotional intelligence” and “social intelligence” are now mainstream notions, stemming from their appealing nature and depiction in popular media. However, empirical attempts at quantifying the quality of social *interactions* have not been nearly as successful as measures of individual differences such as social skills, theory of mind, or social/emotional intelligence. The subjective, lay ratings of the quality of interactions by naïve observers are nonetheless consistent both within and between observers. The goal of this paper is to describe recent empirical work surrounding lay beliefs about social interaction quality and ways in which those beliefs can be quantified. We will then argue that these lay impressions formed about the quality of an interaction, perhaps via affect induction, are consistent with an expertise framework. Affect induction, beginning in infancy and occurring over time, creates instances in memory that accumulate and are ultimately measurable as social-emotional expertise (SEE). The ways in which our lay beliefs about social interaction quality fit the definition of expertise, or the automatic, holistic processing of relevant stimuli, will be discussed. We will then describe the promise of future work in this area, with a focus on a) continued delineation of the thoughts, behaviors, and timing of behaviors that lead to high-quality social interactions; and b) the viability of expertise as the conceptual model for individual differences in social-emotional ability.

## Introduction

Social interactions have long been a source of lay beliefs about the ways in which psychological constructs operate. Indeed, some of the most enduring and popular psychological constructs to become common lay beliefs have originated in research focused on social-emotional interactions. Emotional intelligence (EI) and social intelligence (SI) have both become common parlance due in part to both the intuitive nature of the constructs as well as the success of Daniel Goleman’s popular accounts (Goleman, 1995, 2007). However, the way in which one determines whether a social interaction is positive or negative largely remains very much like Justice Stewart’s dictum: “We know it when we see it.” Empirical attempts at quantifying social interaction quality have not been nearly as successful as measures of individual traits such as social skills (Riggio, 1986), theory of mind (Lawrence et al., 2004), or social and/or emotional intelligence (Silvera et al., 2001; Geher, 2004; Mayer and Salovey, 2007). Although questions remain concerning the latent traits and abilities being measured by the scales, measures of the aforementioned constructs are psychometrically reliable and valid (Thorndike and Stein, 1937; Murphy, 2006; Riggio, 2010). Further, observer reports have been central to the validation of these constructs, with individual differences measures of social traits and abilities routinely compared with observer ratings of the same trait as evidence of the measure’s validity (e.g., Colvin and Funder, 1991; Elfenbein et al., 2015). Despite the common usage of observer report, the subjective, lay interpretation of the quality of an interaction overall *by observers of the interaction* has not been as frequent, though it has been shown to be consistent within and between trained (Alden and Wallace, 1995; Beidel et al., 2010; Glenn et al., 2019) and untrained (Wild and Bachorowski, unpublished) observers.

## Quantifying Lay Beliefs About Interaction Quality

Recent work has shown that ratings of dyadic interaction quality by naïve, third-party observers corresponds with the self-reported social ability of the individuals in the interactions being observed (Wild and Bachorowski, unpublished). These observers watched videos of an interacting dyad. The dyads interacting in the videos were participating in an interview paradigm in which one of the interactants was a trained graduate student using a scripted interview. The interactant was an undergraduate student who completed self-report measures of a variety of social-emotional individual differences, including the Social-Emotional Expertise (SEE) Scale (McBrien et al., 2018). Participants in the observer study were asked to watch the “target” interactant in the interview, the undergraduate student, unaware of the target’s self-reported social ability. They were then asked to rate the quality of the interaction they had just observed. Observers’ ratings were higher for interactions depicting dyads that had higher self-reported social ability, as quantified by the SEE Scale. These results replicated results of a study in which trained “expert” observers, who were involved in social interaction research and given explicit instructions for what to observe in an interaction to determine the overall quality, rated the same set of videos. Like naïve observers, experts rated undergraduate participants with higher self-reported social ability as having higher social interaction quality as well (Wild and Bachorowski, unpublished). Such findings indicate that lay beliefs about the quality of interactions may be a fruitful area for delineating the specific behaviors that promote high-quality social interactions.

Consistent with the findings described above, Elfenbein et al. (2015) demonstrated that observers are able to accurately assess the EI of individuals being observed in social interactions. It is of interest here to note that the self- and other-perception of individuals’ EI was consistent regardless of whether a peer or supervisor was providing the rating. Together, these results indicate that observer perceptions, or lay beliefs, of social-emotional ability can be consistent across social contexts and evaluative judgments. These findings also build on previous work in which dyads rated each other on rapport, and third party observers rated dyads on the same metric (Bernieri et al., 1996; Bernieri and Gillis, 2001). Rapport is a construct related to social ability, but is concerned more with the quality of the dyadic interaction and the behaviors associated with the overall quality, rather than connecting that interaction quality with the specific social ability of individuals in the interaction. By collecting lay observers’ ratings of both the overall interaction quality of a dyad, and participant self-reports of social ability, we are beginning to bridge the gap between lay observers’ interpretations and individual difference metrics that can be used across interaction types.

The findings discussed thus far are based on dyadic interactions. Further work will benefit from studying larger social groupings. Additionally, work investigating the goal of the specific social context will be illuminating. While previous work has considered interacting dyads in a variety of contexts [e.g., interview scenarios (Wild and Bachorowski, unpublished) and collaborative games (Bernieri et al., 1996)], the ratings of interaction quality by lay observers have been consistent. Explicit investigation of whether context shifts lay observers’ interpretations of interaction quality are warranted. Toward this end, recent work has shown that individuals, at least in the cultural context of the United States, rate faces of individuals wearing clothing associated with higher economic status as more competent than faces wearing clothing associated with lower economic status (Oh et al., 2019). These results indicate that factors extrinsic to the specific judgment being made contribute to lay perceptions of an individual’s social characteristics. Further, discrepancies have been identified between individuals’ ratings of subjective success versus objective metrics of success (e.g., Deslauriers et al., 2019). In this example, individuals engaged in active learning reported feeling as though they had performed worse (i.e., learned less) despite performing better on objective metrics of learning. Early evidence does not identify such a discrepancy between self-reported social expertise and lay observers’ ratings of social performance (Elfenbein et al., 2015; Wild and Bachorowski, unpublished). However, to further investigate this potential discrepancy between objective and subjective social performance, more objective, theory-based measures of social performance are required.

## Affect Induction and Social Interactions

As described above, lay observers of social interactions seem to have a good sense of the social ability of the individuals they are observing. These findings do, however, raise an important question: What is the mechanism through which socially able individuals are conveying their ability to others? A recently completed study (Wild and Bachorowski, unpublished) utilized facial electromyography (fEMG) to begin to address the question of mechanism by testing whether individuals observing social interactions experienced greater activity in their zygomatic (associated with positive affect) or corrugator (associated with negative affect) muscles based on the quality of the interaction they were observing. Participants gave continuous ratings of their affect and rated the quality of the interactions they observed. The essential goal of this experiment was to test hypotheses derived from affect induction theory, or the idea that the signals associated with social-emotional behaviors function to elicit affect in others (Owren and Bachorowski, 2003). Prior work investigating the social behavior of laughter has shown that individuals’ use of laughter varies with social circumstances (Owren and Bachorowski, 2003), and that this variability in laugh acoustics differentially drives the amount of positive affect reported by listeners (Bachorowski and Owren, 2001). The results were consistent with an evolutionary perspective on the functional use of affective signals in social interactions (Owren and Bachorowski, 2001). Affect-related signals such as laughter and smiling, as well as other behaviors involved in social interactions (e.g., eye gaze, body position, etc.) would not have adaptive utility if they were simply veridical representations of the internal state of the organism. Instead, it is more parsimonious and consistent with numerous examples from other species (e.g., chameleons shifting color to adapt to the environment and bull snakes mimicking rattlesnakes to avoid predators) where behavior is used to influence the response of observers rather than indicate a true intention (Owren et al., 2003). The argument to be made here is that social animals, chief among them humans, utilize social behaviors to induce affect in others. Including the social partners, themselves, but also the observers of those interactions is consistent with an evolutionary account of social signals (Owren et al., 2003). The recent fEMG study from our group, described above, has yielded results that indicate observers’ affect ratings were indeed impacted by the self-reported social ability of the individuals they were viewing. These results show that lay observers of social interactions are more positively, affectively impacted by the behavior of those with high social ability than those with lower social ability.

## Social-Emotional Expertise (See)

Our ongoing work is focused on testing whether the results described thus far are amenable to an expertise account of individual differences in social interaction ability. In cognitive psychology, expertise is typically defined as the automatic, accurate, and holistic processing of relevant stimuli (Logan, 1985; Gauthier et al., 2000). As such, expertise is domain-specific (e.g., expertise in car identification does not generalize to other domains). A valid and reliable self-report measure of SEE has been developed, with scores related to both convergent and discriminant constructs as predicted (McBrien et al., 2018). As examples, SEE Scale scores are positively correlated with SI, EI, and social skills inventories. These results are an indication of the consistent inter-correlations of measures of constructs involving social behavior and problem-solving, found in meta-analytic reviews, as has been shown in the interpersonal accuracy literature (Schlegel et al., 2017). This work has found that the skills and abilities thought to be associated with interpersonal accuracy, or the ability to correctly judge others’ emotions, intentions, and other social characteristics, are all correlated in the mild to moderate range.

While the fact that interpersonal accuracy was determined by Schlegel et al. (2017) to be a collection of separate, mildly to moderately correlated skills and abilities is important, there are further implications. Expertise in any given domain requires a similar constellation of moderately correlated skills and abilities, not all of which are necessary, but several of which are sufficient (e.g., Richler et al., 2019). For instance, it is not necessary to have high fluid intelligence to be a car expert, however, there is a correlation between fluid intelligence and visual ability and performance on a visual expertise task (Sunday et al., 2018a). The combination of skills, abilities, and acquired experience necessary for expertise is not a fixed ratio, and the skills and experiences necessary are not strictly limited. It is therefore plausible that expertise in the social domain occurs in much the same way, with a set of moderately correlated skills and abilities available to an individual to utilize as components of their social expertise. If this is the case, the level of social expertise, or the successful utilization of social skills and abilities, is the true individual differences metric. This is not to claim that other metrics of individual differences in social skills and abilities are not informative. Those metrics are important, and may provide the specific skills and abilities that comprise a given individual’s social expertise. Below, we outline how lay impressions of social ability and these more specific metrics of social skills inform the central aspects of our nascent expertise account of individual differences in social ability.

## Automaticity in Social Interactions

Our proposal that social interactions might operate as a domain of expertise is consistent with the instance theory of automaticity, which states that a skill becomes more automatic as more instances of successful completions of the skill accrue, and therefore is more readily accessible in memory (Logan, 1988, 1995). The more instances present in memory, the greater the probability of accessing an instance of that skill (or action) quickly, thereby promoting automaticity. In the context of affect induction in social interactions, increased instances of both inducing affect in others and having it induced in one’s self could ultimately produce smooth, automatic affect-related responses.

Instances are formed each time a skill is utilized, and become more automatic as a result of practice (Logan, 1997). Context influences skill success, so the context of a skill’s utilization is important. For instances of successful social skill, social context must be taken into account, and is likely variable across cultures, socio-economic statuses, race/ethnicities, gender identities, and more. It follows that SEE would vary with experience in a given social context, just as has been found for other social-emotional individual difference measures (e.g., Rimé et al., 1990; Oh et al., 2019). More experience with interactions in one culture would lead to more automatic, and therefore expert, performance in that culture than in a culture with which experience is limited. This has been demonstrated in emotion recognition, a process that is automatic and yet varies based on in-group/out-group experience (e.g., Elfenbein and Ambady, 2003; Beaupré and Hess, 2006; Hess and Fischer, 2014). Baseline SEE may influence the starting point of competence in navigating novel social situations. In the same way that a car expert will more readily learn to identify a novel vehicle than a novice, so too might someone high in SEE learn to adapt to novel social contexts than someone lower in the SEE spectrum. In this way a SEE framework for individual differences in social ability can account for differences in social ability within and between cultures as a function of baseline ability and experience. This application of automaticity to instances of social interactions also provides an account for how friendships can build over time, as two people accrue instances of successful interactions across shared contexts. The social exchanges seen between close friends, so often described in lay observation as “effortless,” may in fact be automatic.

## Holistic Processing in Social Interactions

A second hallmark of expertise frameworks for skills in other domains, such as car experts or radiologists, is the holistic processing of relevant visual stimuli (Richler et al., 2011). Holistic processing can be measured in various ways. One demonstration of holistic processing involves focusing on the overall stimulus and then very quickly honing in on only the most relevant details for making an informed decision. Holistic processing is exemplified by radiologists looking first at an entire CT image before narrowing in quickly on the parts of the image that are consistent with lesions (Wood, 1999; Sheridan and Reingold, 2017), or car experts first looking at the car as a whole before zeroing in on the aspects that identify the unique make and model of the vehicle (Gauthier et al., 2000; Sunday et al., 2018b). Further, these visual ability metrics and fluid intelligence are both associated with successful performance on a visual detection task in radiological images (Sunday et al., 2018a) and performance on these tasks has been linked with specific activation in the fusiform face area, an area associated with expertise (Gauthier et al., 1999; Tarr and Gauthier, 2000; Gauthier and Nelson, 2001; Sunday et al., 2018b). In much the same way, individuals in a social interaction may need to see the overall state of the interaction before zeroing in on specific aspects to improve the outcome of the interaction, and this process may require a combination of social interaction ability and SI and/or EI. The visual expertise for faces may instead reflect the role faces play in human social interactions and exemplify the way in which individuals acquire and adapt social ability as an expertise. Impairment in holistic processing of a social interaction could impair social interaction quality, such as the impairments in interaction quality seen in Social Anxiety Disorder (SAD). Individuals with SAD have long described being too focused on specific behaviors in the interaction (usually their own) to focus on the overall interaction. This focus on specific behaviors then leads to difficulty tracking the needs of the other person in the interaction, thereby negatively affecting the overall quality of the interaction (Mueller et al., 2009). To frame this clinical disorder as being in part attributable to an error of holistic processing, one’s focus on a specific behavior (e.g., “what am I doing with my hands”) precludes the ability to focus on the overall interaction, leading to an impairment in both holistic processing of the social-emotional stimuli and the automatic processing of relevant stimuli. Stepping back, this brief description of SAD is illustrative of a lay characterization creating a scientifically viable framework for research.

## Summary and Further Directions

Lay beliefs about the quality of social interactions and the ways in which those interactions can be impaired are proving important for building a scientific description and explanation of social-emotional interactions. The lay belief that the “chemistry” between two people, rather than the attributes of just one participant in an interaction, is vital to its success has been borne out in the data we have collected. Observers’ ratings of a dyad’s interaction quality are not related to traits unique to one person, but to the ability, or expertise, of each individual at adapting to others successfully. The lay beliefs of those with social anxiety have identified impaired holistic processing as a key component in the disruption of their social interaction quality. Further work will focus on a continued delineation of the thoughts and behaviors that lead to high-quality social interactions and viability of expertise as the conceptual model for individual differences in social-emotional ability. Such a model will also allow for the development of objective measures of social performance that can answer questions regarding the consistency between subjective and objective ratings of social performance. As this delineation continues, it will be crucial to not lose sight of the ways in which lay beliefs offer ecological validity. Lay beliefs about social interactions could be an essential guide to our elucidating the mechanistic underpinnings of human interaction.

## Data Availability Statement

The datasets generated for this study are available on request to the corresponding author.

## Ethics Statement

The studies involving human participants were reviewed and approved by the Vanderbilt University Institutional Review Board. The patients/participants provided their written informed consent to participate in this study.

## Author Contributions

Both authors contributed to the conceptualization and construction of this manuscript.

## Conflict of Interest

The authors declare that the research was conducted in the absence of any commercial or financial relationships that could be construed as a potential conflict of interest.
